# *Pseudomonas aeruginosa* PA80 is a cystic fibrosis isolate deficient in RhlRI quorum sensing

**DOI:** 10.1038/s41598-021-85100-0

**Published:** 2021-03-11

**Authors:** Syed A. K. Shifat Ahmed, Michelle Rudden, Sabrina M. Elias, Thomas J. Smyth, Roger Marchant, Ibrahim M. Banat, James S. G. Dooley

**Affiliations:** 1 School of Environment and Life Sciences, Independent University, Bangladesh (IUB), Dhaka, Bangladesh; 2 Department of Biology, University of York, Wentworth, York, YO10 5DD UK; 3 School of Science, Institute of Technology Sligo, Sligo, Ireland; 4 School of Biomedical Sciences, Ulster University, Coleraine, UK

**Keywords:** Microbiology, Microbial genetics

## Abstract

*Pseudomonas aeruginosa* uses quorum sensing (QS) to modulate the expression of several virulence factors that enable it to establish severe infections. The QS system in *P. aeruginosa* is complex, intricate and is dominated by two main *N*-acyl-homoserine lactone circuits, LasRI and RhlRI. These two QS systems work in a hierarchical fashion with LasRI at the top, directly regulating RhlRI. Together these QS circuits regulate several virulence associated genes, metabolites, and enzymes in *P. aeruginosa*. Paradoxically, LasR mutants are frequently isolated from chronic *P. aeruginosa* infections, typically among cystic fibrosis (CF) patients. This suggests *P. aeruginosa* can undergo significant evolutionary pathoadaptation to persist in long term chronic infections. In contrast, mutations in the RhlRI system are less common. Here, we have isolated a clinical strain of *P. aeruginosa* from a CF patient that has deleted the transcriptional regulator RhlR entirely. Whole genome sequencing shows the *rhlR* locus is deleted in PA80 alongside a few non-synonymous mutations in virulence factors including protease *lasA* and rhamnolipid *rhlA, rhlB, rhlC.* Importantly we did not observe any mutations in the LasRI QS system. PA80 does not appear to have an accumulation of mutations typically associated with several hallmark pathoadaptive genes (i.e., *mexT, mucA, algR, rpoN, exsS, ampR*). Whole genome comparisons show that *P. aeruginosa* strain PA80 is closely related to the hypervirulent Liverpool epidemic strain (LES) LESB58. PA80 also contains several genomic islands (GI’s) encoding virulence and/or resistance determinants homologous to LESB58. To further understand the effect of these mutations in PA80 QS regulatory and virulence associated genes, we compared transcriptional expression of genes and phenotypic effects with isogenic mutants in the genetic reference strain PAO1. In PAO1, we show that deletion of *rhlR* has a much more significant impact on the expression of a wide range of virulence associated factors rather than deletion of *lasR*. In PA80, no QS regulatory genes were expressed, which we attribute to the inactivation of the RhlRI QS system by deletion of *rhlR* and mutation of *rhlI.* This study demonstrates that inactivation of the LasRI system does not impact RhlRI regulated virulence factors. PA80 has bypassed the common pathoadaptive mutations observed in LasR by targeting the RhlRI system. This suggests that RhlRI is a significant target for the long-term persistence of *P. aeruginosa* in chronic CF patients. This raises important questions in targeting QS systems for therapeutic interventions.

## Introduction

Quorum sensing (QS) is a cell density dependent signal transduction mechanism used by prokaryotes to regulate population level gene expression^1^. *Pseudomonas aeruginosa* has a sophisticated QS network that is orchestrated by three main signalling pathways namely *las*, *rhl* and *pqs*^2,3^. Each of these pathways are composed of a synthase protein that produces signal molecules called autoinducers (AI). AI molecules bind to the corresponding receptor proteins, this AI-ligand bound receptor complex regulates several genes in bacteria^4^. Two of the most well-studied QS systems in *P. aeruginosa* are the acyl homoserine lactone based *las* and *rhl* systems. The autoinducer synthase LasI catalyses the formation of the autoinducer *N*-(3-oxododecanoyl)-L-homoserine lactone (C_12-_HSL) which binds to the transcription regulator LasR to form a transcriptional activator complex (LasR:C_12_-HSL) that regulates the expression of QS regulatory genes *rhlR* and *pqsR* of the *rhl* and *pqs* pathways, respectively^5–7^. The transcription regulator RhlR forms a complex with the *rhlI* catalysed product *N*-butanoyl-L-homoserine lactone (C_4_-HSL). The RhlR:C_4-_HSL complex binds to conserved *rhl* sites in the promoter regions of the target genes including *rhlI* to trigger a second autoinduction-forward loop^8,9^. In studies with standard laboratory strains like PAO1 and PA14, mostly in nutrient rich medium, LasR has been shown to directly induce *las*, *rhl* and *pqs* systems thereby often being considered as the de facto QS regulator controlling QS mediated virulence in *P. aeruginosa*^5,10,11^*.*

QS regulated virulence is mediated by multiple factors including pyocyanin, rhamnolipid and exopeptidases^12^. Pyocyanin, one of the main phenazines found in CF patients has redox activity that can increase the production of reactive oxygen (ROS) species thereby producing hydrogen peroxide with detrimental effects to the host cell^13^. Exopeptidases like elastase have been shown to degrade opsonizing lung surfactant proteins^14^ and inactivate antimicrobial peptides LL-37^15^. Proteases have also been shown to cause damage to the lung epithelial lining and the degradation of complement proteins, fibrinogen and immunoglobulins^16–18^. Another widely studied QS metabolite, rhamnolipid, has been shown to cause necrosis of human polymorphonuclear leukocytes^19^, to support bacterial twitching and swarming motilities^20,21^ and to maintain biofilm architecture^22,23^. All these QS regulated metabolites are essential to early establishment of *P. aeruginosa* infection in lungs^24–26^ with mutations in QS phenotypes becoming common as the *P. aeruginosa* adapts to their host environment during chronic stages of infection^27–29^.

The importance of QS has also been demonstrated in transmissible lineages of *P. aeruginosa*. The hypervirulent Liverpool epidemic strain (LESB58) was shown to upregulate and overproduce QS phenotypes, potentially contributing for enhanced morbidity and successful spread of the LES lineage throughout the CF population in the UK^30,31^. Therefore, with QS being strongly associated with virulence in clinical outcomes, ongoing research has been investigating alternative methods to attenuate bacterial virulence, especially now when antibiotics are a limited resource. A promising alternative to antibiotics has been in the use of anti-QS compounds such as trans-cinnamaldehyde (CA) and salicylic acid (SA)^32^ which reduce expression of QS associated virulence factors by targeting the master regulator, LasR, in *P. aeruginosa*^33,34^. Although these LasR anti-QS inhibitors have worked well with laboratory strains by decreasing the QS mediated virulence factor expression^35,36^, its effect on isolates collected from chronic patients has not been fully validated. There is a growing consensus of LasR mutations are frequent among chronic clinical isolates^29,37,38^. These LasR mutants were shown to induce exaggerated host inflammatory response, neutrophil degradation and immunopathology in animal models^38^. Paradoxically, it has been shown that in these LasR mutants, the QS hierarchy has shifted such that RhlR QS is independent of direct LasR regulation^39^. These findings therefore question how rigidly maintained is the established QS hierarchy in *P. aeruginosa* and whether LasR is the best target in anti-QS strategies.

In this study we have characterised a clinical isolate of *P. aeruginosa* that is deficient in RhlRI QS. RhlR mutants are very rarely isolated from CF patients and are mostly associated with hypermutability^28^. To understand the effect of this RhlR mutant on QS regulation and virulence gene expression, we have compared this isolate (PA80) with isogenic QS mutants in *P. aeruginosa* PAO1. We show that PA80 is completely deficient in expression of QS regulatory genes and QS regulated virulence genes. PA80 is closely related to the hypervirulent Liverpool epidemic strain LESB58. We attribute the complete loss of virulence expression and QS related activity to the inactivation of the RhlRI system as PA80 does not maintain any non-synonymous mutations in common pathoadaptive genes typically mutated in *P. aeruginosa* CF isolates. PA80 is an example of the diversity of long-term pathoadapted *P. aeruginosa* CF isolates.

## Results

### *P. aeruginosa* CF isolate PA80 is a RhlRI mutant

**Figure 1:**
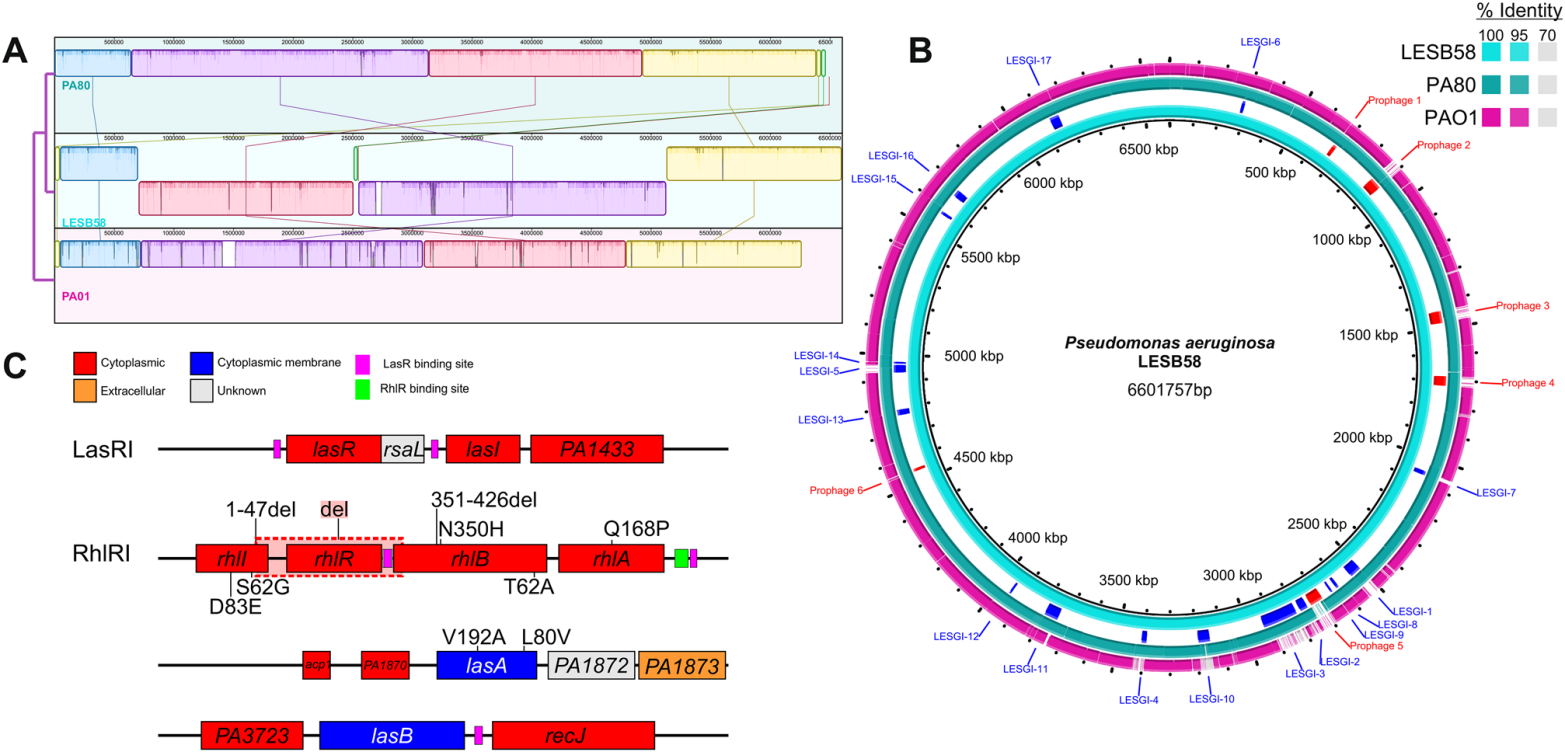
Comparative genomics of PA80. (**A**) Whole genome alignment of PA80, PAO1 and LESB58. PA80 shares 99.53% sequence identity to LESB58. (**B**) Whole genome comparison of PA80 with LESB58 and PAO1. LESB58 genomic islands are highlighted in blue and prophage region in red. Coordinates were mapped to LESB58 genome from Jani et al., 2016^88^. PA80 contains only one unique region relative to LESB58. PAO1 lack several of the GI’s and prophages identified in LESB58. (**C**) Genomic context of the main non-synonymous mutations in PA80. Shaded red indicates complete deletion of the *rhlR* gene.

**Figure 2:**
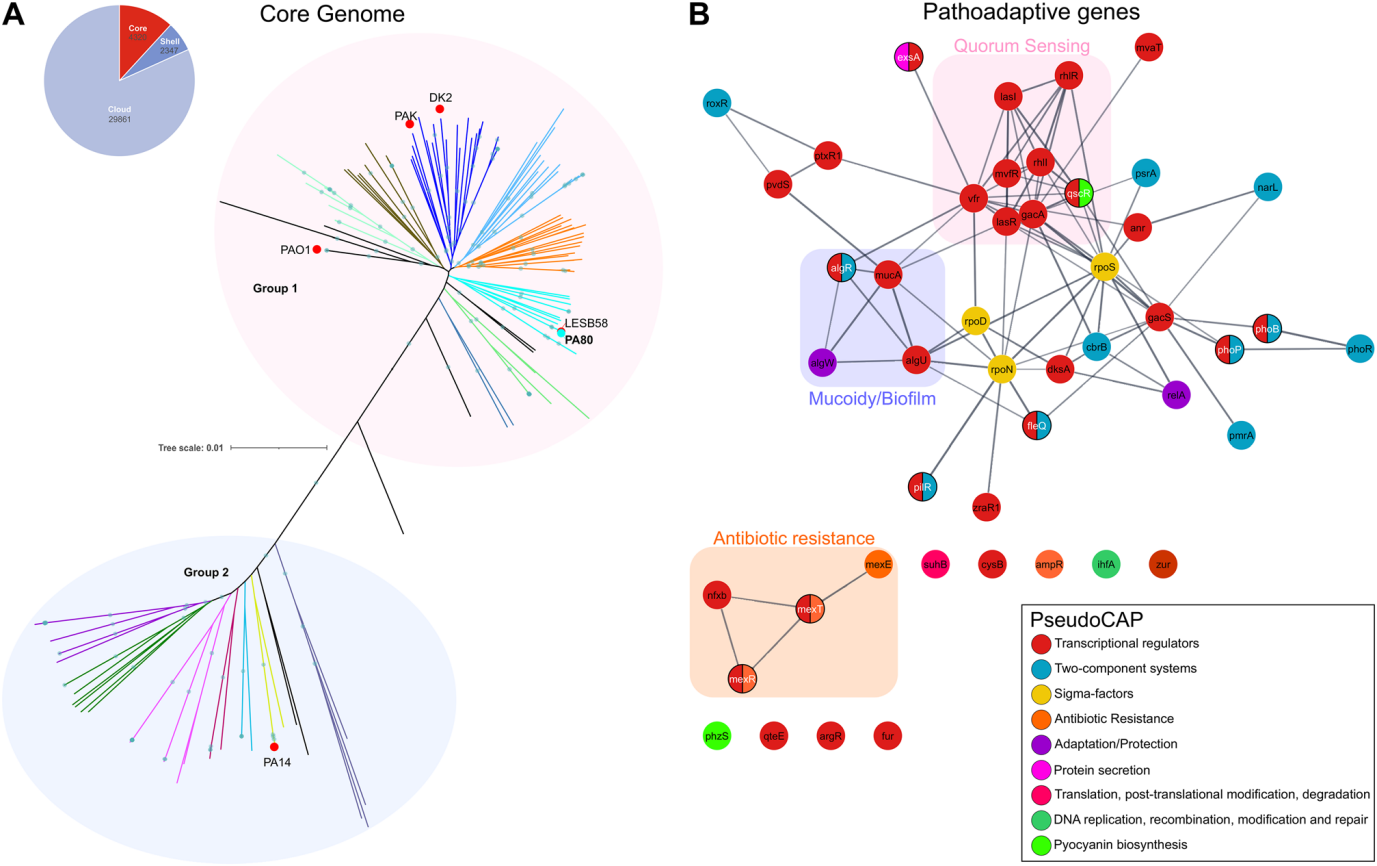
Core genome phylogeny of *P. aeruginosa*. (**A**) Maximum likelihood phylogenetic tree of *P. aeruginosa* core genome. PA80 clusters with hypervirulent Liverpool Epidemic strains LESB58 and LES431. The widely referenced PAO1 strain is distinctly separate from more virulent strains that have been isolated from Cystic Fibrosis patients. Important *P. aeruginosa* strains are highlighted by red dot, strain PA80 is indicated by a cyan dot. The number of genes determined in the core across 95% of the *P. aeruginosa* isolates are shown inset in the pie chart. (**B**) A STRING interaction network to show common pathoadaptive genes (n = 47) that can be mutated in *P. aeruginosa* isolates from CF patients. Genes are colored based on Pseudocap annotations. Note—the following proteins are not found in the STRING database *vqsR, rsaL, pprB*, PA4851, PA152, and are not shown in the network.

Whole genome sequencing (WGS) was carried out on *P. aeruginosa* isolate PA80, a clinical isolate collected from the sputa of a Cystic Fibrosis patient attending Regional Cystic Fibrosis Centre in Northern Ireland^24^. de novo assembly of PA80 generated 97 contigs, the genome size of PA80 is 6,500,365 bp with an average G.C content of 66.39%, similar to other published *P. aeruginosa* genomes. Whole genome alignment (WGA) showed that PA80 is 91.26% identical to the reference strain PAO1 and 90.74% similar to PA14. However, a high similarity was found between PA80 and *P. aeruginosa* Liverpool epidemic strain LESB58 with a 99.53% sequence match (Fig. 1B). PA80 lacks the large inversion (~ 4.5 Mb) observed in LESB58 (Fig. 1A). To understand the lineage of PA80 we performed a pan-genome analysis in comparison with 265 publicly available genomes from *Pseudomonas*.com. The core genome was determined using the core gene alignment output from roary. Core genome phylogeny reveals two distinct groups of *P. aeruginosa,* similar to previously published studies^40–43^. PA80 is found in a clade with LES isolates LESB58 and LES431 in both the core and accessory genome (Fig. 2A and Fig. S1).

Figure 2B highlights a list of common pathoadaptive genes that are known to mutate in *P. aeruginosa* CF isolates during adaptation and evolution to the lung environment. In PA80 we identified several SNPs, most of which are synonymous SNPs found in these common functional pathoadaptive mutations among *P. aeruginosa* (Table 1). For a more extensive list please see Table S1. However, the main feature of PA80 is the deletion of the *rhlR* gene. The entire *rhlR* gene has been deleted along with the C-terminal of *rhlB* and the N-terminal of *rhlI* (Fig. 1C). The core genome is generally conserved with sequence diversity only ranging between 0.5 and 0.7%^44^. *Pseudomonas aeruginosa* diversification arises from the accessory genome which is reflected in the varying genome sizes ranging from 5.5 to 7 Mbp^45^. PA80 accessory genome shares > 99% sequence identity with LESB58 and contains several LES genomic island and prophage regions (Fig. 1B). PA80 is larger in genome size compared to PAO1 indicative of a larger accessory genome (Fig. S1A). Phylogeny of the accessory genomes shows that PA80 is most closely related to LESB58 (Fig. 2A). Functional annotation using RAST^46^ revealed that the region contained 15 genes involved in phage replication, packaging, capsid proteins and lysis; 5 genes involved with copper transport system and 11 genes responsible for resistance to antimicrobial and toxic compounds like cobalt, zinc and mercury (Fig. S1B). This suggests that PA80 is a derivative of the *P. aeruginosa* LES lineage and to the best of our knowledge this is the first report of a LES *rhlR* mutant isolated from a CF patient.

**Table 1:** PA80 genetic variations relative to PAO1.

Gene	Function	SNPs	Mutation type	Ref
**Regulatory genes**				
*lasR*	Global regulator of quorum sensing circuit, involved with expression of factors which gives bacteria many of its pathogenic trait	0	–	^89^
*lasI*	Produces a key autoinducer signal molecule C12HSL which positively regulates QS	0	–	^90^
*rhlR*	Global regulator of the *rhl* mediated QS		Deletion	^6^
*rhlI*	Produces a key autoinducer signal molecule C4-HSL which positively regulates QS	8	S62G, D83E	^91^
*mvfR*	Involved in production of QS signal molecules and can regulate multiple QS controlled genes without affecting the *las* or the *rhl* QS systems	1	Synonymous	^92^
*rsaL*	Global regulator that represses *lasI* transcription and functions in opposite to LasR by counterbalancing C12-HSL concentrations	0	–	^93^
*vfr*	A global regulator that induces expression of the *lasR* promoter and virulence gene expressions	1	Synonymous	^94^
*ampR*	It plays a dual role, positively regulating the *lasB*, and *rhlR* expression levels and negatively regulating the *lasA*, *lasI*, and *lasR* expressions	1	Synonymous	^95^
*dksA*	Inhibits QS virulence factor productions by repressing transcription of *rhlI*	0	–	^96^
*suhB*	A positive global regulator of *P. aeruginosa* virulence genes	6	Synonymous	^97^
*pilR*	Transcriptional regulator of piliation- associated with virulent phenotype motility	9	E318D	^98^
*mexT*^a^	Involved with increased antimicrobial resistance and repression of QS	14	Synonymous	^99^
*mexS*^a^	Involved with increased antimicrobial resistance through activation of the mexEF-oprN operon	4	D249N, M271I	
*vqsR*	Activated by *lasQS* and plays essential role in acyl-HSL production	1	Synonymous	^100^
*qteE*	Represses the expression of several *las* and *rhl*-dependent target genes by independently reducing LasR and RhlR protein stability	3	Synonymous	^101^
**Two component regulatory system**				
*gacA*	Positively controls QS through activation of the Rhl system	3	Synonymous	^102^
*gacS*	Regulates QS by controlling the expression of *rsmY* and *rsmZ*	7	Synonymous	^102^
*pmrA*	Modulates resistance to cationic antimicrobial peptides	5	L71R, D104Y	^103^
*phoR*	Involved with induction of virulence genes in low phosphate conditions	4	Q58H	^51^
*phoB*	Regulates cytotoxicity through modulation of QS systems in low phosphate conditions	0	–	^51^
*pprB*	Positively regulates transcription of type I secretion system, components, fimbriae and type IV pili	9	S129N, R179K, P191S	^104^
**Sigma factors**				
*rpoN*	Regulates the expression of *rhlI* and *pqsR*	2	Synonymous	^105^
*rpoS*	Regulates expression of pyocyanin, exotoxin, LasA and LasB elastases etc	3	Synonymous	^106^
*rpoD*	It recognizes a large number of promoters and controls expression of housekeeping genes	3	Synonymous	^107^
*pvdS*	Involved in expression of pyoverdine and exotoxin A; also functions as iron starvation sigma factor	0	–	^108^

^a^PA80 MexT and MexS sequences were aligned with PA14.

### A functional autoinducer is essential for QS gene expression

**Figure 3:**
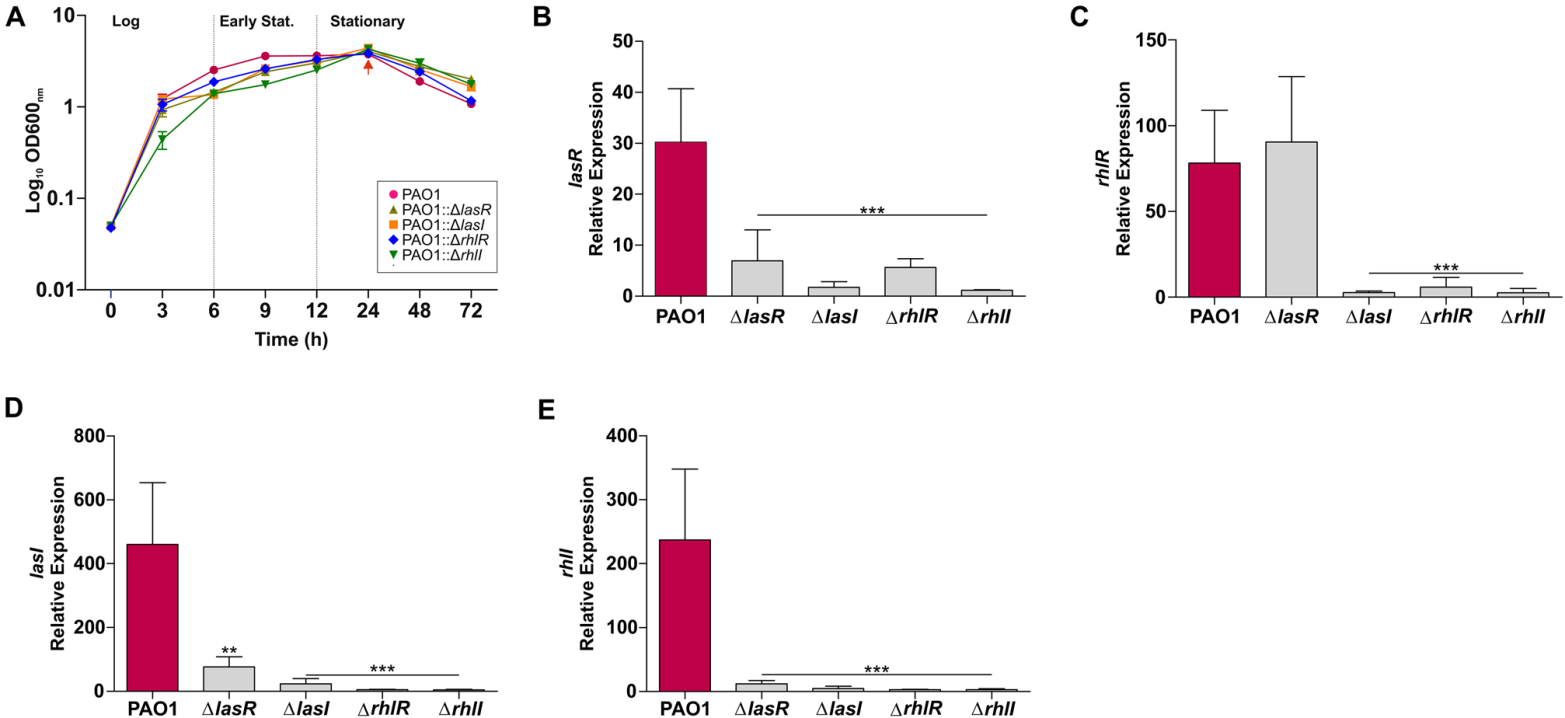
Inactivation of QS regulatory genes reduces both *las* and *rhl* QS systems. (**A**) Growth phenotypes of PAO1 and isogenic QS regulatory mutants in a phosphate limited media (PPGAS). Relative expression levels of QS regulatory genes (**B**) *lasR,* (**C**) *rhlR,* (**D**) *lasI,* and (**E**) *rhlI* in both PAO1 and QS mutants. Relative expression levels were quantified in the stationary phase (indicated by red arrow) of growth by qRT-PCR. Error bars represent S.D. ± (n = 3 biological replicates). All mutant data was analysed relative to PAO1 expression values. Significance was determined by a one-way ANOVA followed by Dunnett’s multiple comparison test (*p < 0.05, **p < 0.01, ***p < 0.001).

To understand the role of RhlRI in *P. aeruginosa* QS, we investigated individual isogenic mutants in both the LasRI and RhlRI QS systems in the reference strain PAO1. Inactivation of either *las* or *rhl* QS does not significantly affect the growth of PAO1 under phosphate limiting conditions (Fig. 3A) except for a slight lag in *ΔrhlI*. It has been reported that the *rhl* system can work independently of *las* regulation through the observation that LasRI null mutants are frequently isolated from CF patients^29,39,47^.To assess if these systems can work independently, we examined the gene expression of QS regulatory and virulence associated genes using the PAO1 QS mutants. We show inactivation of RhlRI abolishes expression of both *las* and *rhl* regulatory genes (Fig. 3B–E). However, inactivation of *lasR* did not affect expression of RhlR (Fig. 3C) demonstrating that RhlR expression can be independent of LasRI. Deletion of either LasRI and RhlRI systems can be complemented by restoring a functional protein or in the case of the synthases protein (*lasI* and *rhlI*) by exogenous autoinducer molecules^48,49^. Recent studies have elegantly demonstrated evolutionary trajectories for LasR null mutants where a functional RhlRI system independent of LasRI quickly emerges^48–50^. This was an interesting finding considering the QS hierarchy puts *lasR* at the top of the QS system with the LasRI activated complex inducing expression of the *rhlR* regulator. However, we do note that phosphate limitation is known to induce expression of key QS regulatory genes. Our results support recent work by Meng et al., 2020 which report increased expression of *rhlR* in a LasR mutant strain of PAO1 under phosphate-depleted conditions^51^. Similar to our results this increased expression was not observed for *rhlI* (Fig. 3E). Meng et al., 2020 show this increased expression is due to activation by the PhoR/PhoB two- component regulatory system^51^. The unaltered *rhlR* expression in the absence of a functional *lasR* therefore challenges the established QS hierarchy and further supports recent claims of other regulatory pathways capable of inducing *rhl* QS, by bypassing the *las* QS system in a nutrient deprived environment^50^. However, in *ΔlasR,* expression of *rhlI* was completely switched off to levels observed for *ΔrhlI* and all other QS mutants (Fig. 3E).

**Figure 4:**
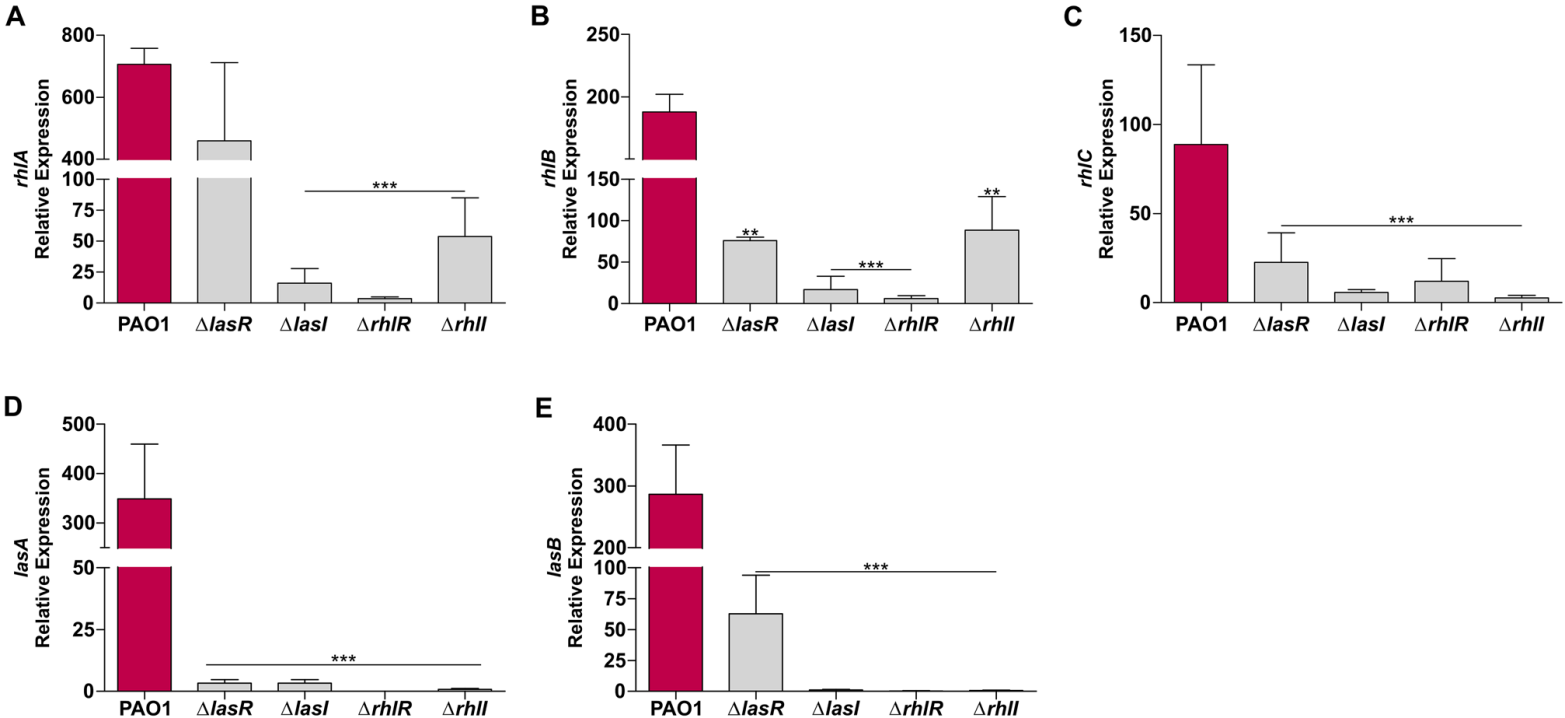
Inactivation of LasRI and RhlRI QS systems reduces virulence factor expression. Relative expression levels of virulence factor genes (**A**) *rhlA,* (**B**) *rhlB,* (**C**) *rhlC,* (**D**) *lasA* and (**E**) *lasB*. Relative expression levels were quantified in the stationary phase (indicated by red arrow) of growth by qRT-PCR. Error bars represent S.D. ± (n = 3 biological replicates). All mutant data was analysed relative to PAO1 expression values. Significance was determined by a one-way ANOVA followed by Dunnett’s multiple comparison test (*p < 0.05, **p < 0.01, ***p < 0.001).

To understand the effect of knocking out the different QS systems, we measured the expression of virulence genes under the control of either *las* or *rhl*. Rhamnolipids are low molecular weight glycolipids that play important role in *P. aeruginosa* pathogenesis^52^. RLs are synthesised de novo in *P. aeruginosa* by the biosynthetic genes *rhlABC* that are directly regulated by the RhlRI QS system^53^. *rhlAB* is not expressed in *ΔrhlR* whereas inactivation of *ΔlasR* does not affect their transcriptional expression (Fig. 4A,B). Expression of the protease LasA and the elastase LasB is positively regulated by LasR^54^. We show expression of *lasA* is completely abolished in all QS regulatory mutants (Fig. 4D). Expression of *lasB* was downregulated in *ΔlasR* but not completely inhibited as observed in the other mutants (Fig. 4E). It is well established that RhlR mediated activity can be uncoupled from LasR regulation by the isolation of LasR mutants that are RhlRI active^28,39^. However, we note that signal negative mutants (*ΔlasI ΔrhlI*) have a significant effect on expression of QS regulated genes (Fig. 4). Expression of *lasA*, *lasB* and *rhlABC* is completely inhibited in signal negative *ΔlasI* and significantly reduced in *ΔrhlI* (Fig. 4). This suggests that production of the signal molecules may be the most critical part for a functional QS system.

### Deletion of RhlR inhibits QS activity in clinical isolate PA80

**Figure 5:**
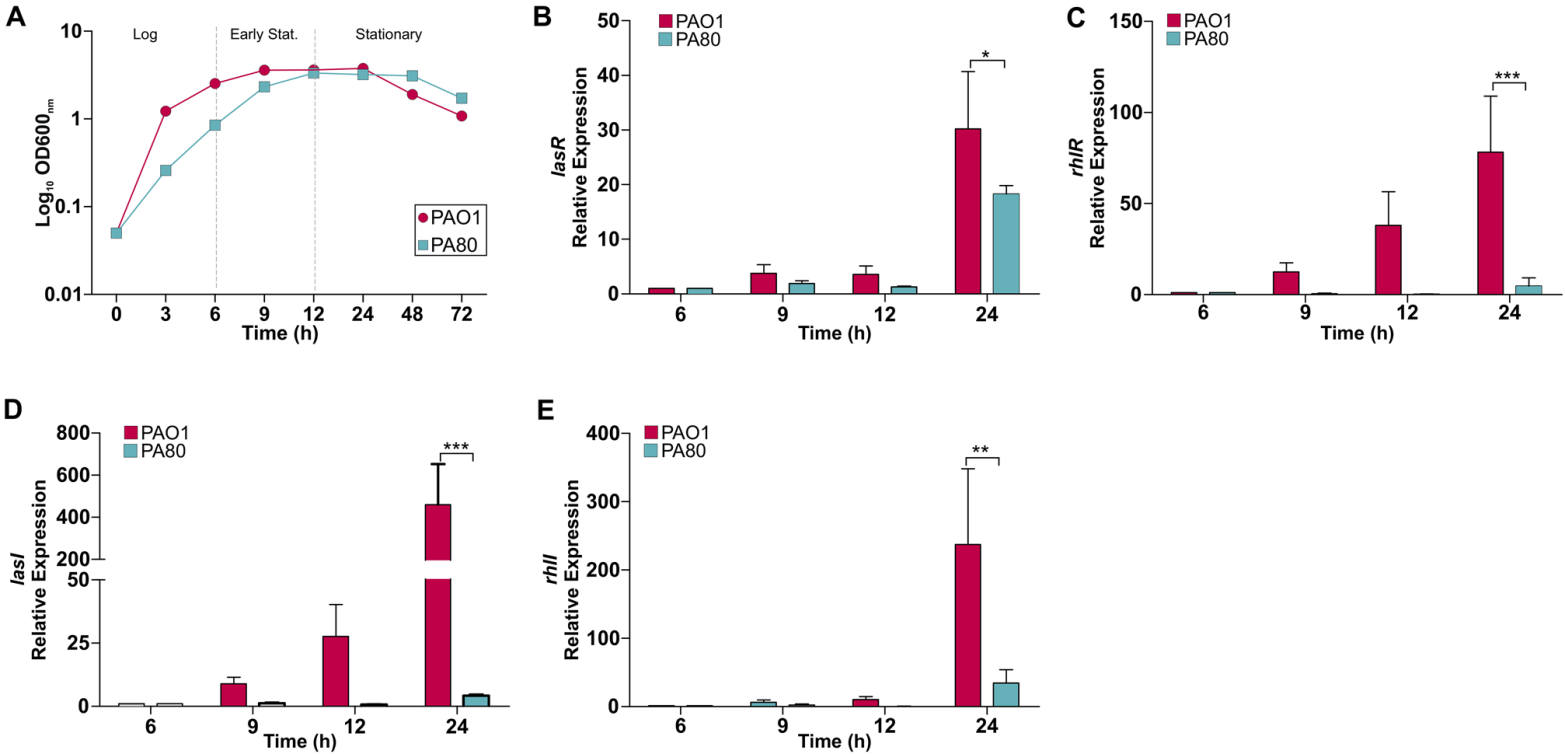
CF isolate PA80 does not express any QS regulatory genes. (**A**) Growth of PAO1 in comparison with *rhlR* mutant isolate PA80. Relative expression of PAO1 and PA80 during log and stationary growth phases. Relative expression levels of QS regulatory genes (**B**) *lasR,* (**C**) *rhlR* (**D**) *lasI* and (**E**) *rhlI* in both PAO1 and PA80. Expression levels are shown as the mean relative expression ratios to log phase levels (i.e. 6 h). Error bars represent the S.D (biological triplicates). Data was analysed using a one-way ANOVA followed by a Dunnett’s multiple comparison test comparing each time point to log phase levels (i.e. 6 h) (*p < 0.05, **p < 0.01, ***p < 0.001).

**Figure 6:**
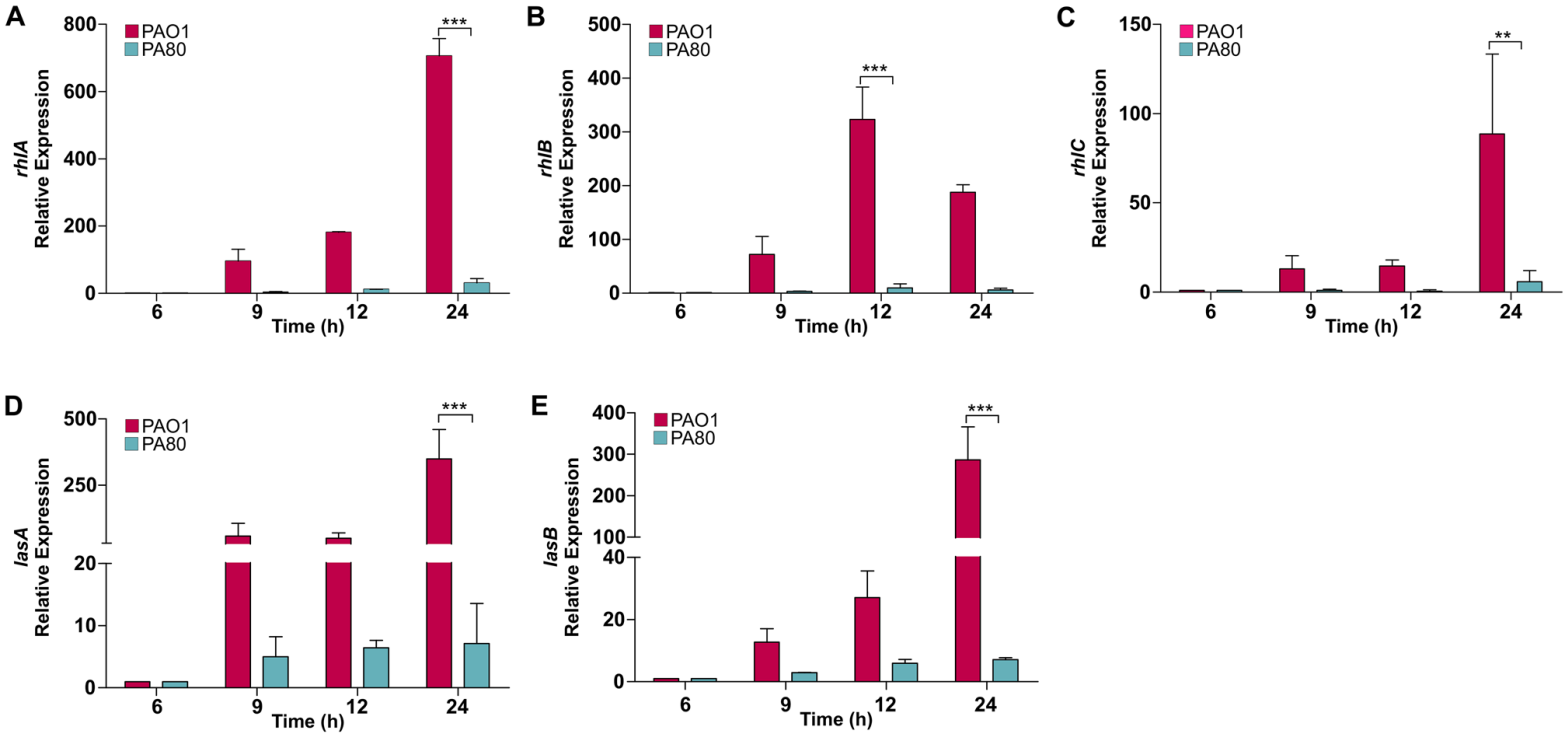
Repression of QS genes attenuates virulence factor production in PA80. Relative expression levels of QS regulated virulence genes (**A**) *rhlA,* (**B**) *rhlB,* (**C**) *rhlC* and (**D**) *lasA* and (**E**) *lasB* in both PAO1 and PA80. Expression levels are shown as the mean relative expression ratios to log phase levels (i.e. 6 h). Error bars represent the S.D (biological triplicates). Data was analysed using anone-way ANOVA followed by a Dunnett’s multiple comparison test comparing each time point to log phase levels (i.e. 6 h) (*p < 0.05, **p < 0.01, ***p < 0.001).

We also determined the effect of the *rhlR* deletion in PA80 by examining the expression of QS regulatory genes and virulence gene expression. Similar to the signal negative *ΔrhlI,* the clinical isolate PA80 too showed a significant delay in log phase growth compared to PAO1 (Fig. 5A). This could suggest that a functional *rhl* system is important for the growth of *P. aeruginosa.* PAO1 shows a typical AHL-dependent expression of QS genes, where expression correlates with an increase in cell density (Fig. 5). As expected, we observe expression of *lasR* in PA80 during the stationary phase similar to PAO1 (Fig. 5B). The expression of both *rhlR* and *rhlI* was completely abolished in PA80 (Fig. 5C,E). Interestingly we did not detect expression of *lasI* in PA80 (Fig. 5D), this was surprising as no mutations were detected in *lasI* or upstream in the promoter region (Table 1). In correlation with the lack of QS gene expression in PA80, the virulence factor expression was completely switched off (Fig. 6) while PAO1 expressed the virulence associated genes in a typical growth phase dependent manner (Fig. 6)^32^.

### Inactivation of QS attenuates virulence factor production

**Figure 7:**
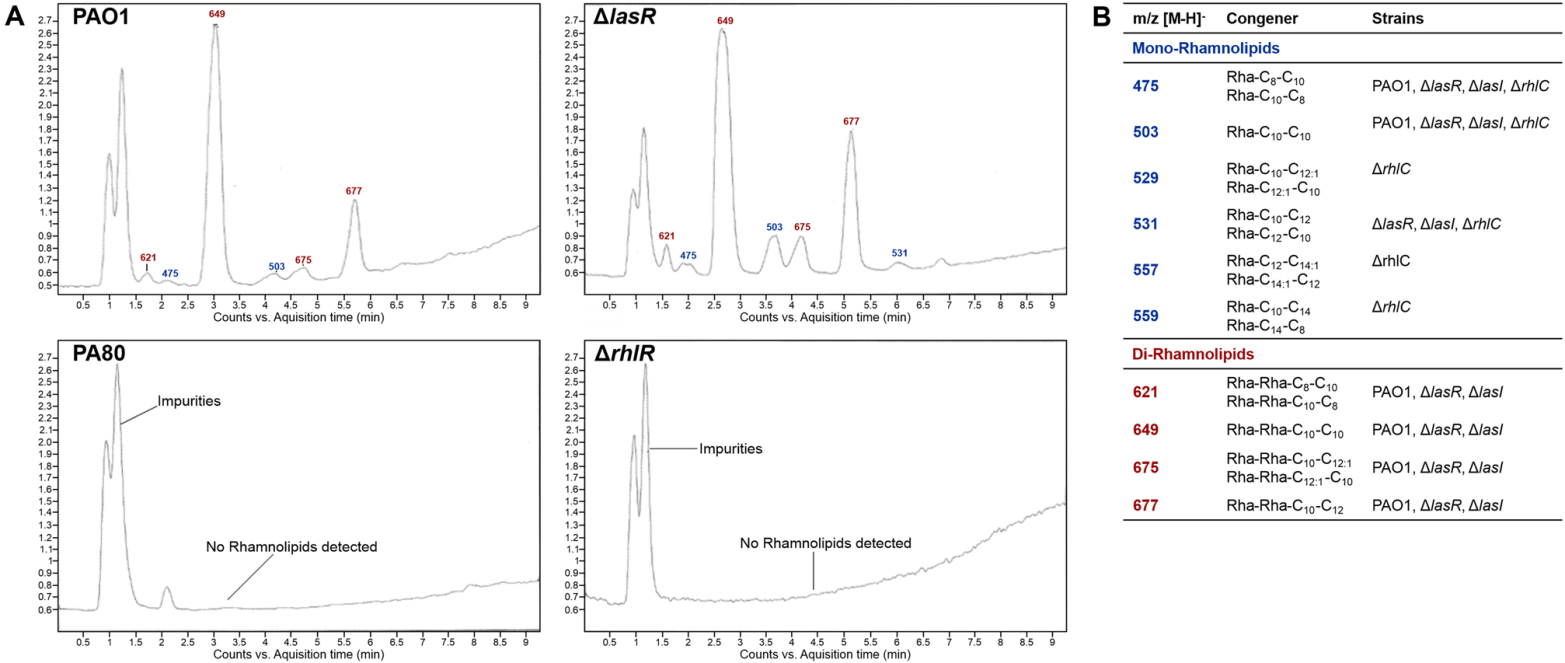
PA80 does not produce rhamnolipids. (**A**) HPLC–MS chromatogram of rhamnolipids (RLs) detected in PAO1, *ΔlasR, ΔrhlR* and PA80. Rhamnolipids are produced independent of *lasR* but it is essential to have a functional *rhlR* present. PA80 lacking *rhlR* does not produces RLs. (**B**) Comparison of the RLs congeners produced in wild-type PAO1 and mutant strains. *ΔlasR* produces RLs congeners in the same composition as PAO1.

**Figure 8:**
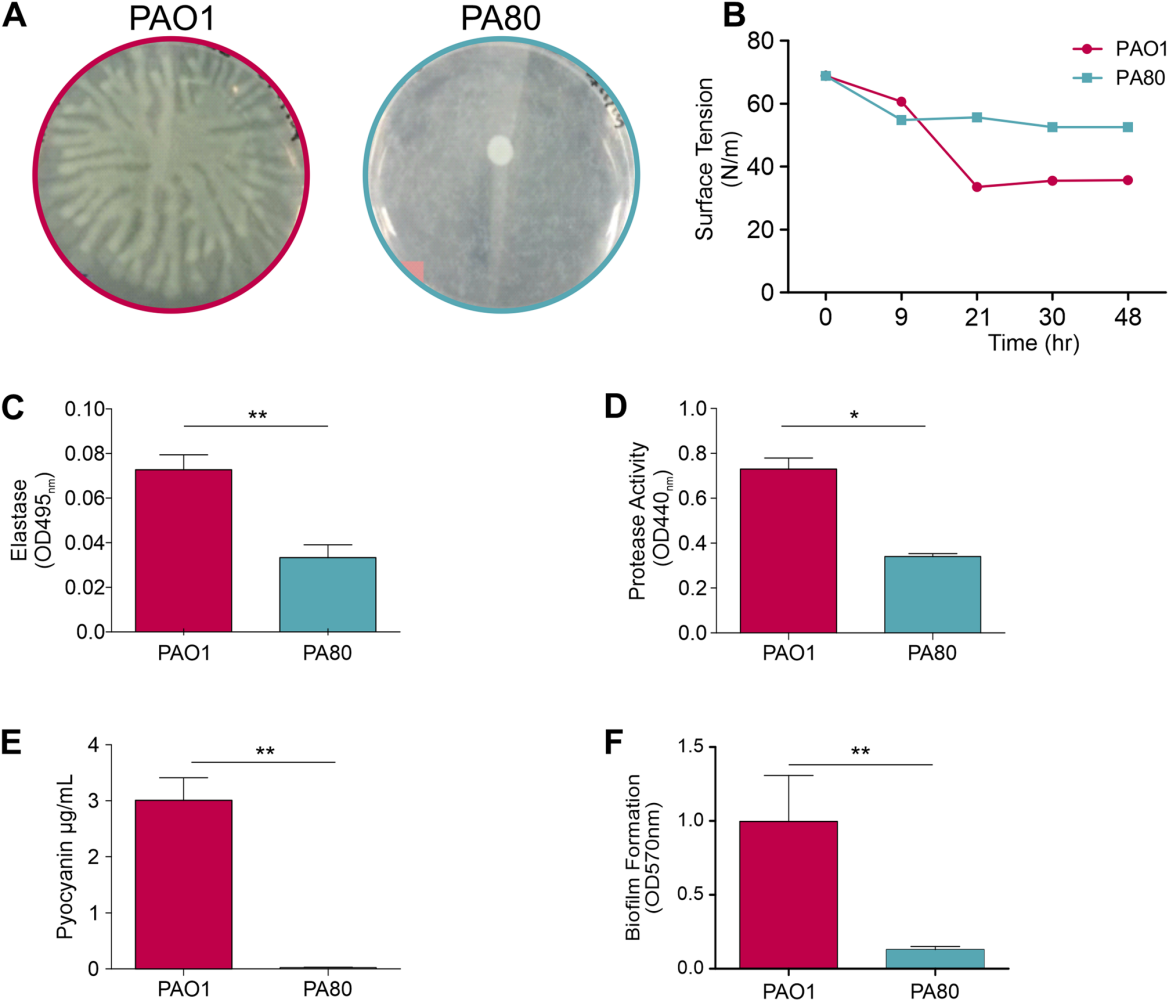
PA80 has reduced virulence factor production. (**A**) Swarming motility of PAO1 and PA80. (**B**) PA80 does not reduce surface tension due to lack of RLs produced. PAO1 typically reduces surface tension of water to ~ 38 (N/m). Production of (**C**) elastase (**D**) protease (**E**) pyocyanin and (**F**) biofilm in PAO1 and PA80.

We measured the production of extracellular virulence factors in both PAO1 and PA80 (Figs. 7, 8). In correlation with the lack of rhamnolipid gene expression, PA80 does not produce any rhamnolipids (Fig. 7). Production of extracellular rhamnolipids was quantified by HPLC–MS from crude extracts for both PAO1 and PA80. We also quantified rhamnolipid production in both QS and *rhlABC* mutants (*ΔlasR, ΔlasI, ΔrhlR, ΔrhlI, ΔrhlA, ΔrhlB and ΔrhlC*). PAO1 produced both mono- and di-rhamnolipids with the di-rhamnolipid congeners Rha-Rha-C_10_-C_10_ (m/z 649) and Rha-Rha-C_10_-C_12_ (m/z 677) being most abundant (Fig. 7A). Production of rhamnolipids is under the control of the *rhl* QS system, inactivation of *lasRI* does not affect extracellular production of rhamnolipids (Fig. 7A). However, inactivation of *rhlR* abrogates rhamnolipid production in both PA80 and *ΔrhlR*. While di-RLs are predominant, its production is dependent on the conversion of mono-RLs to di -RLs. Therefore, when we delete *rhlC,* which is responsible for di-RL production, we only detect mono-RL congeners (Fig. 7B). These data clearly support that production of RLs is stringently regulated in *P. aeruginosa*^55^ and *rhlR* is essential for its production.

Similar to other pathoadapted *P. aeruginosa* isolates, PA80 has reduced production of several virulence factors^56^. RLs are essential for motility in *P. aeruginosa*, as expected PA80 exhibits no motility (Fig. 8A). Consistent with the lack of RL production in PA80, is the inability to decrease the surface tension of water (Fig. 8B). We also show that PA80 does not produce virulence associated secretions for elastase, protease and pyocyanin (Fig. 8C–E). PA80 is also a weak biofilm producer in comparison to PAO1 (Fig. 8F). Loss of these virulence phenotypes has been reported for both LasR^−ve^ and RhlR^−ve^ mutants, Chen et al., 2019 show that deletion of *lasR* in PAO1 and mutation of RhlR in strain E80 abolishes pyocyanin and protease production^49^. Similar phenotypes have been reported Kostylev et al., 2020 with LasR mutants^50^. Here we show loss of function for both *las* and *rhl* controlled virulence factors even though PA80 maintains a functional LasRI system, thereby suggesting that RhlRI could be the linchpin in the *P. aeruginosa* QS hierarchy.

## Discussion

A hallmark of *P. aeruginosa* evolution in the CF lung is loss of function in key virulence associated phenotypes such as quorum sensing (QS). It is now well established that during chronic CF infections, *P. aeruginosa* can rewire its QS hierarchy such that LasR, the prime regulator that directly or indirectly controls the expression of other key regulators in the QS pathway is no longer functional^49^. This enables RhlR to act independently of LasR regulation and makes its vital for long term survival within the host^49^. LasR null mutants are commonly isolated from chronic CF infections^27,29,39,57,58^, these isolates typically have attenuated virulence but maintain an active RhlRI QS system^59,60^. As a result, LasR was considered a viable therapeutic target for anti-virulence drugs^61^. Emergence of LasR mutants in *P. aeruginosa* CF isolates is regarded as evidence for adaptive evolution to the CF lung environment^56^ however the benefit of these mutants still remains unclear. It has been suggested that these loss of function in QS mutants may have an advantage in nutrient acquisition in the CF lung environment, act as social cheats or maybe a functional QS system is only required for establishing infection^62^. Regardless, isolation of LasR mutants in *P. aeruginosa* are frequent among chronic CF infections, whereas mutations in RhlRI are not common with only a few reported cases from very late stages of infection^28^.

Here we report a *P. aeruginosa* isolate PA80 that is a RhlR null LasR^+ve^ mutant. Based on phylogeny we propose that PA80 belongs to the divergent lineage of the Liverpool epidemic Strain (LES) (Fig. 1), the most common strain to infect the CF population in the UK^63^. PA80 shares 99.53% core genome identity with LESB58. Functional annotation of the accessory genome in PA80 revealed the presence of several prophage genes which have been linked to enhanced competitiveness and fitness of LESB58^63^. These prophage elements are known to be important for early stage infection^63,64^ while also being significant reservoirs for horizontal gene transfer (HGT) of several antimicrobial resistant determinants which is characteristic among *P. aeruginosa* strains^65^. The unique feature of PA80 is that this strain is a highly pathoadapted LES variant that has completely inactivated its QS system due to the deletion of the *rhlR* locus.

WGS identified several mutations in PA80, most of which were synonymous mutations among several key global regulators (Table 1). It is interesting to note, PA80 contains a predicted functional MexT by the clean deletion of the duplicated 8 bp sequence found in PAO1 (Table 1). In several PAO1 lineages the presence of this duplicated 8 bp produces a truncated MexT polypeptide (89aa) which lack six terminal residues of the HTH DNA binding domain, thus in *P. aeruginosa* strains containing this 8 bp duplication, the MexEF-OprN operon is not expressed^66^. PA80 also contains the common MexS-D_249_N mutation observed in other clinical *P. aeruginosa* isolates^67^. Most of the mutations observed in PA80 are synonymous with no major deleterious or loss of function effects with the exception of the targeted deletion of the *rhlR* locus. To date, the paradigm for *P. aeruginosa* evolution in the CF lung has more or less adhered to the following pathway: (1) initial colonisation of wild type *P. aeruginosa*, (2) emergence of LasR^−VE^ cheats with RhlRI functionality via mutation of *mexT*, (3) PQS null mutants in LasR^−VE^ MexT^−VE^ isolates. PA80 is a late-stage chronic CF isolate, it represents a distinct pathoadapted variant of LES in that it does not contain any of the expected genotypes as mentioned above, rather the *rhlR* locus seems to be targeted for complete deletion. Not only are RhlR mutants rare, they are typically restricted to late stage chronic CF infections and strongly correlate with precursor *lasR* mutations^28^. RhlRI is critical for the regulation of several virulence associated phenotypes that are required for colonisation and acute infection in CF patients^48^.

One of the most striking features of PA80 is that there are no mutations associated in the *lasR-lasI* genomic region, whereas the *rhlR* locus has been targeted for deletion alongside the N-terminal of *rhlI* and C-terminal of *rhlB* (Fig. 1). To understand the loss of function in RhlRI QS in PA80, we initially analysed PAO1 isogenic QS mutants for expression and production of QS regulated phenotypes. Consistent with previous reports of RhlR QS independent of LasR regulation we observed expression of *rhl* regulated rhamnolipid and elastase genes in *ΔlasR*. We show that expression of the virulence factors rhamnolipids and elastase are not affected by inactivation of LasR. However, we do note that inactivation of the AHL signal gene *lasI* had a significant impact on both QS regulatory and virulence gene expression in PAO1. Much less attention has been focused on inhibition of QS signal molecules as a therapeutic target in comparison to LasR. However, as we learn more about the intricate and complex network of QS in *P. aeruginosa* it is obvious that LasR is not a viable therapeutic target therefore research has now shifted to RhlR as a potential target^68,69^.

We observe complete downregulation of *LasRI* in PA80 even though there are no mutations in this genomic region or with any associated global regulators that drive expression of LasR (Table 1). This correlated with the downregulation of *lasB* which is stringently regulated by the *las* system^70,71^. This suggests that inactivating the RhlRI system has significant impact on LasRI expression. We also show this in the isogenic QS mutants in wild-type PAO1, inactivation of either *rhlR* or *rhlI* represses expression of both *lasRI* (Fig. 3). We do not know the mechanism by which inactivation of RhlRI in PA80 has abolished LasRI activity. There could be unknown regulatory elements that maybe upregulated or repressing expression of *lasRI*—however this does suggest that PA80 is a highly pathoadapted strain that has loss of function in both *las* and *rhl* QS systems. In several LasR mutants isolated from the CF lung environment, the QS hierarchy seems to be readily reprogrammed such that RhlRI independent from LasR can be achieved by simple genetic changes in the global regulator *mexT*^49,72^. While it seems typical to rewire the QS circuitry such that RhlRI is independent of a functional *las* system, the inverse however does not seem to be true.

Recently Chen et al. 2019^49^ demonstrated in vitro by experimental evolution that RhlR mutants do not readily emerge in LasR^−VE^ mutants, rather mutations emerge in the non-AHL *Pseudomonas* quinolone signal (PQS) and the related 2-alkylquinolone (HHQ) molecules. Chen et al., 2019 also showed that constructed RhlRI null mutants have a significant fitness cost that is outcompeted by the wild-type and are completely sensitive to cyanide which is synthesised by *P. aeruginosa*. Production of cyanide in *P. aeruginosa* is considered a metabolic policing mechanism by which it monitors cheaters (mutant in public goods) which are typically more susceptible to cyanide toxicity in comparison to wild-type^73^. We also observe a significant fitness cost in PA80 when grown in phosphate limiting peptide rich media (Fig. 5). Interestingly we also show that *ΔrhlI-*PAO1 has a similar growth defect in comparison to wild-type PAO1 (Fig. 3). This suggests that RhlRI is essential in *P. aeruginosa* even in heterogenous populations that undergo rapid evolutionary changes during CF infection and evolution. A functional RhlRI system is necessary to allow for the emergence of LasR cheats, where such strains become dependent on C_4_-HSL secreted by cells with QS intact, as a result of this dependence mutations are less likely to arise in RhlRI. However, this selection pressure is most likely observed during the early stages of infection where *P. aeruginosa* undergoes rapid microevolution. Late-stage chronic infections are characterised by high genotypic and phenotypic diversity, that is reflective of highly adapted lineages that persist long term. These variants have attenuated virulence, that helps evade immune recognition and enables long term *P. aeruginosa* persistence and survival^74^.

Our study is significantly limited to the examination of a single isolate and standard laboratory growth conditions compared to the dynamic selection pressures and polymicrobial conditions of the CF lung environment. However, we can glean some significant insights to *P. aeruginosa* evolution from PA80. PA80 was isolated from late-stage chronic infection, we however do not know anything about the evolutionary dynamics that selected for the deletion of the *rhlR* locus. It is unlikely that PA80 would emerge in isolation, most likely there would have been a divergent clonal population with several mutant populations/cheats (i.e. LasR^−VE^, LasR^−VE^MexT^−VE^) to enable the emergence of cheats while in the presence of other QS active wild-types and intermediates.

Nonetheless, PA80 provides a unique evolutionary trajectory which, to our knowledge has not been reported to date. This is an important discovery as the focus shifts from developing inhibitors that target LasR to RhlR. Our data show the loss of function *rhlR* does render PA80 avirulent in both *las* and *rhl* regulated virulence. This taken together with the fact that a functional RhlRI is essential during early infection and cannot be easily rewired as seen with LasR, RhlR may be a better therapeutic target. However, mutants can arise in RhlR, therefore targeted inhibition should be aimed at early-stage acute infections rather than in long term chronic infections. PA80 provides another genome available for comparison of long term pathoadapted *P. aeruginosa* isolates from the CF lung. In the CF lung *P. aeruginosa* undergoes an evolutionary pathway that can take several directions, however as we build a better genomic map of these adaptations, it is clear that a multi-target approach is needed to treat the highly divergent *P. aeruginosa* lineages.

## Materials and methods

### Bacterial strains and growth media

The clinical isolate, PA80 was obtained from the culture bank maintained at the Ulster University in Coleraine campus. The isolate was initially collected from a cystic fibrosis patient (aged 20) attending the CF clinic in the Belfast City Hospital^24^. All the *P. aeruginosa* PAO1 mutants (*ΔlasR, ΔlasI, ΔrhlR, ΔrhlI, ΔrhlA, ΔrhlB, ΔrhlC*) were purchased from the *P. aeruginosa* mutant library maintained at Manoil Laboratory in the University of Washington^75^. The well-studied and fully sequenced *P. aeruginosa* PAO1 was used as the control strain in the experiments. *Pseudomonas aeruginosa* PAO1 is also QS proficient^32^. All the overnight cultures were prepared from the − 80 °C frozen culture stocks either in a LB or nutrient broth and cultivated under at 37 °C with shaking at 180 rpm. The overnight culture was used to inoculate the phosphate limited proteose-peptone-glucose-ammonium-salts (PPGAS) medium^76^ for growth and gene expression experiments. All experimental reagents were purchased from the Sigma-Aldrich, UK unless mentioned otherwise. Experiments performed in biological triplicates.

### Nucleic acid extraction and quality-check

Genomic DNA: gDNA was isolated from a freshly prepared overnight culture using the Wizard Genomic DNA Purification Kits (Promega) following the manufacturer’s protocol. The Nanodrop 1000 spectrophotometer (Thermo Fisher Scientific) was used for DNA quantification and purity assessment. High quality DNA (A_260/280_ and A_260/230_ ~ 1.8 and ~ 2.0 respectively) were only considered for further experiments and were stored in small aliquots in nuclease free water at − 20 °C.

Total RNA: The cell pellets from the different bacterial cultures were collected at the experimental time points for RNA isolation using the JetGene RNA Purification Kit (Thermo Fisher Scientific). In brief, the cells were lysed in a buffer solution containing 1X TE buffer, 20 mg/ml proteinase K (Promega) and 15 mg/ml lysozyme. The lysed samples were then transferred to a 2 ml Lysing Matrix A tube (MP Biomedicals) containing RLT buffer from the kit and β-Mercaptoethanol (10 µl/ml). The contents in the Matrix A tube were homogenised using the FastPrep FP 200 cell disrupter at speed 5.5 for 30 s. Following centrifugation, the supernatant was transferred to RNeasy spin columns (Qiagen) for DNase treatment. Another round of lysing buffer treatment was performed before doing a second DNase treatment. The RNA extracted was quantified and assessed for purity similarly to DNA. The integrity of the RNAs isolated were ascertained through visualization of two sharp bands corresponding to 16S and 23S rRNA under UV light following electrophoretic separation on agarose gel. Additionally, the RNA samples were also checked for integrity with Agilent 2100 Bioanalyser.

### Reverse transcription quantitative polymerase chain reaction

500 ng of target mRNA was added to a reaction mix consisting of 20–250 ng of random primers (Promega) and 10 mM dNTPs (Invitrogen). The reaction was incubated for 5 min at 65 °C for cDNA synthesis. After the incubation, the mix was centrifuged briefly and 5X stand buffer, 0.1 M DTT and RNase out (Invitrogen) were added in volumes corresponding to final concentrations of 1X, 10 µM and 40 units respectively. A second incubation at 25 °C for 2 min was performed before addition of Superscript II Reverse Transcriptase (200U final concentration). This was followed by a series of incubation steps: 25 °C for 10 min, 42 °C for 50 min and 70 °C for 15 min to give the first strand cDNA. The cDNA synthesis was performed for all biological replicates. A negative control without reverse transcriptase was added in every run. All newly synthesized cDNAs were stored at − 20 °C prior to use as template for real time PCR amplification.

Real time qPCR was performed with the ROCHE LightCycler LC480 system using SYBR-Green. Before the mRNA transcripts were quantified, the qPCR primers for the target genes were validated for specificity by generating a PCR calibration curve using PAO1 gDNA. For the mRNA quantification study, only those primers that gave a slope value of − 3.1 to − 3.6 and amplification efficiencies of 90–110% were selected. The primers binding specificity was further confirmed by the presence of a single sharp peak in the melt curve.

Each of qPCR reaction mix contained 2X SYBR Green master mix, 1 µM of forward and reverse primers, cDNA template and nuclease free water to make up the 10 µl volume. Negative controls in form of no reverse transcriptase (NRT) and no template DNA (NTD) and positive control in form of gDNA were included for accuracy. Cut-off values for residual gDNA and negative controls were set at greater than 35 and 40 cycles respectively. The qPCR amplification conditions used were: initial denaturation for 5 min at 95 °C, 40–50 cycles of denaturation for 10 s at 95 °C, annealing for 10 s at 59 °C, extension for 10 s at 72 °C.

### Relative gene expression data analysis

The reference gene validation and selection were done using six candidate genes (*gyrB, proC, cysG, rpoD, rpoB* and *16S*). Three different and independent software packages were used to select for the most stable genes as previously reported from our lab^32^. Based on the algorithms of these programs, the candidate genes *rpoD* and *proC* were selected as the most stable genes for use as reference genes in *P. aeruginosa* PAO1.

System (LC480 software, version 2) generated analysis was performed on the real time qPCR data following the steps outline by Ahmed et al. (2019)^32^. In brief, relative quantities (RQ) values were calculated using the threshold values (Cq) of the technical replicates. The RQ values of the target genes were divided by the geomean of the reference genes to generate the normalised relative quantity values (NRQ). The relative expression value at the early log (6 h) analysis was used as experimental calibration value to calculate the relative expression of the target genes at the different experimental time points for plotting.

### QS virulence factors quantification

Overnight cultures of PAO1 and PA80 were grown in PPGAS medium for 24 h. Cell-free supernatants were collected through centrifugations, and filter sterilised for use in the following assays:

*LasA protease* Amount of protease production in the culture was assessed by incubating the reaction mixture containing 0.1 ml of the supernatant and 0.8% azocasein (in 500 µl of 50 mM K_2_HPO_4_) at 25 °C for 3 h. The reaction was stopped by first adding 0.5 ml of 1.5 M HCl and then cooling it on ice for 30 min. The tube was centrifuged, and the supernatant transferred in a fresh tube. 1 N NaOH was added to the collected supernatant in equal volumes and the concentration of the acid soluble azopeptides was measured at 440 nm using a UV–vis spectrophotometer.

*LasB elastase* In this assay, 2 ml reaction buffer containing 100 mM Tris-HCl, 1 mM CaCl_2_ and the enzyme substrate elastin-congo red was incubated with 1 ml of the overnight culture supernatant for 3 h at 37 °C for 3 h at 180 rpm. The reaction was stopped by first adding 2 ml of 0.7 M sodium phosphate buffer (pH 6) and then cooling it on ice for 15 min. The mixture was centrifuged, and the supernatant collected for spectrophotometric measurement of the congo-red dye released due to elastase activity in the supernatant at 495 nm.

*Pyocyanin* In a 50 ml tube, 7.5 ml of the culture supernatant was mixed vigorously with 4.5 ml of chloroform till the colour changed to greenish-blue. The mixture was spun and 3 ml of the resulting blue/pink colour solution from the bottom layer was transferred to a fresh tube containing 1.5 ml of 0.2 M HCl. The tube was vortexed vigorously and the resulting pink colour solution was collected for spectrometric measurement at 520 nm. The concentration (µg/ml) of pyocyanin was calculated as OD X 17.072^77^.

### HPLC–MS analysis of rhamnolipid production

Estimation through surface tension reduction ability: 15 ml of the cell free culture supernatants were collected from the different experiment time points of growth. The surface tension was measured using the Du Nouy ring method with a digital tensiometer (Kruss, K10ST, Hambury, Germany)^78^. The ability of the supernatant to reduce the surface tension of the medium is indicative of the presence of the surface-active reducing agent rhamnolipid.

High-Performance Liquid Chromatography Mass Spectrometry/Mass Spectrometry (HPLC MS/MS): At first, crude rhamnolipid was extracted following the protocol outline by Smyth et al. (2010)^79^. Briefly, the cell-free supernatant from the different cultures were collected and acidified with 32% HCl to pH ~ 2. The acidification made the rhamnolipid less soluble in the aqueous state by causing protonation. The acidified supernatant was shaken vigorously in a separating funnel with equal amount of ethyl acetate until two distinct layers became visible; the aqueous layer containing unwanted compounds and the ethyl acetate organic layer containing the rhamnolipid. The rhamnolipid containing organic phase was dried with anhydrous MgSO_4_ and then filtered to collect the filtrate in a round bottomed flask. The organic solvent was evaporated in a rotary evaporator (Buchi, Flawil, Switzerland) to leave a yellowish oily residue containing the crude rhamnolipid.

The crude rhamnolipid was purified using solid phase extraction by passing the samples through a conditioned Strata SI-1 Silica (55 µm, 70A) Giga tubes (Phenomenex). Solvent mixture of chloroform and methanol in 5:0.3 ratio was passed through the column to elute the mono-rhamnolipids from the samples. The same mix again but in 5:0.5 ratio was now passed to elute the di-rhamnolipids from the samples, leaving any remaining impurities trapped in the column.

The pure rhamnolipid extract was analysed for congener composition using an LCQ quadrupole ion trap with a negative ESI interface linked to a Thermofisher spectra system HPLC as explained earlier by Ahmed et al., (2019)^32^.

### Genome assembly and comparative genomics

The PA80 whole genome sequence was provided by MicrobesNG (http://www.microbesng.uk) which is supported by the BBSRC (grant number BB/L024209/1). The PA80 gene sequence has been submitted to GenBank (PRJNA675745) and is now publicly available. The PA80 genomic DNA library was prepared using Nextera XT Library Prep Kit (Illumina, San Diego, USA) with slight modifications. Hamilton Microlab STAR automated liquid handling system was used for DNA quantification and library preparation. The pooled libraries were quantified using the Kapa Biosystems Library Quantification Kit for Illumina on a Roche light cycler 96 qPCR machine and sequenced on the Illumina HiSeq using a 250 bp paired end protocol. Reads were adapter trimmed using Trimmomatic 0.3v software and for de novo assembly SPAdes v3.7 was used. The total number of contigs in the PA80 genome assembly was 97. The number of contigs of length ≥ 0 bp and length ≥ 1000 bp were 145 and 90 respectively. The assembled contigs were then annotated and aligned with the reference PAO1 genome (GCF_000006765.1) using BWA-MEM^80^ and variant calling was performed using VarScan and annotated using Prokka 1.11. Only for MexT and MexS, the PA14 (GCF_006974045.1) genome was used for reference as previously recommended^67^. From the genome sequences, using NCBI local blast (BLAST v2.10) the specific gene sequences were extracted, and alignments were compared. Using the BAM alignment file generated by BWA-MEM algorithm, variants like SNP, insertion and deletions were identified using Mega-X software.

From the mapping statistics it was found that a large portion (8.74%) of the raw reads remained unmapped. This was performed using BWA-MEM tool with *P. aeruginosa* PAO1 as the reference. Hence from the alignment files the unmapped reads were extracted and was assembled into contigs using the spades^81^ tool. The contigs were then aligned against the NCBI nucleotide database using the BLASTN tool and was found to match mostly with the *Pseudomonas aeruginosa* LESB58 genome. The assembled contigs were annotated using RAST^46^ and was functionally annotated using the associated SEED viewer^82^. Later a de novo assembly was generated using the raw reads using spades and it was annotated using RAST and SEED viewer. In addition, the raw reads were also aligned with the publicly available LESB58 (GCF_000026645.1) and PA14 reference genome using bwa^80^ and samtools^83^ to generate mapping statistics. Whole genomes were aligned using MAUVE multiple genome alignment. *Pseudomonas aeruginosa* genomes were downloaded from NCBI assembly. Genome assemblies were annotated with PROKKA and provided as input to Roary. Pangenome analysis was carried out using Roary version 3.12.0^84^. Roary was run using default parameters except for the following: -e -n (to produce alignments with MAFFT) and -i 95. The accessory genome phylogeny was visualised in iTOL using the accessory_binary_genes_fa.newick file output from Roary. Genomic islands and prophages were predicted with IslandViewer^85^ and PHASTER^86^ respectively. BLAST Ring Image Generator (BRIG)^87^ was used to compare *P. aeruginosa* genomes and visualise mobile genetic elements.

## Supplementary Information

Supplementary Information
