# Supplementary material for: Pseudomonas aeruginosa PA80 is a cystic fibrosis isolate deficient in RhlRI quorum sensing
Source: Sci Rep. 2021 Mar 11;11:5729. doi: 10.1038/s41598-021-85100-0 (PMC7970962; doi:10.1038/s41598-021-85100-0)
Supplement: Supplementary file 1 — Supplementary Information [file 41598_2021_85100_MOESM1_ESM.pdf]

Pseudomonas aeruginosa PA80 is a Cystic Fibrosis isolate deficient in RhlRI quorum sensing.

Syed A. K. Shifat Ahmed[†a], Michelle Rudden[†b], Sabrina M. Elias[a], Thomas J. Smyth[c], Roger Marchant[d], Ibrahim M. Banat[d] and James S.G. Dooley[d*]

[†]These authors contributed equally to this work.
[a] School of Environment and Life Sciences, Independent University, Bangladesh (IUB), Dhaka, Bangladesh
[b]Department of Biology, University of York, Wentworth, York, YO10 5DD, UK
[c]School of Science, Institute of Technology Sligo, Ireland
[d]School of Biomedical Sciences, Ulster University, Coleraine, UK,
*correspondence: jsg.dooley@ulster.ac.uk

SUPPLEMENTARY INFORMATION

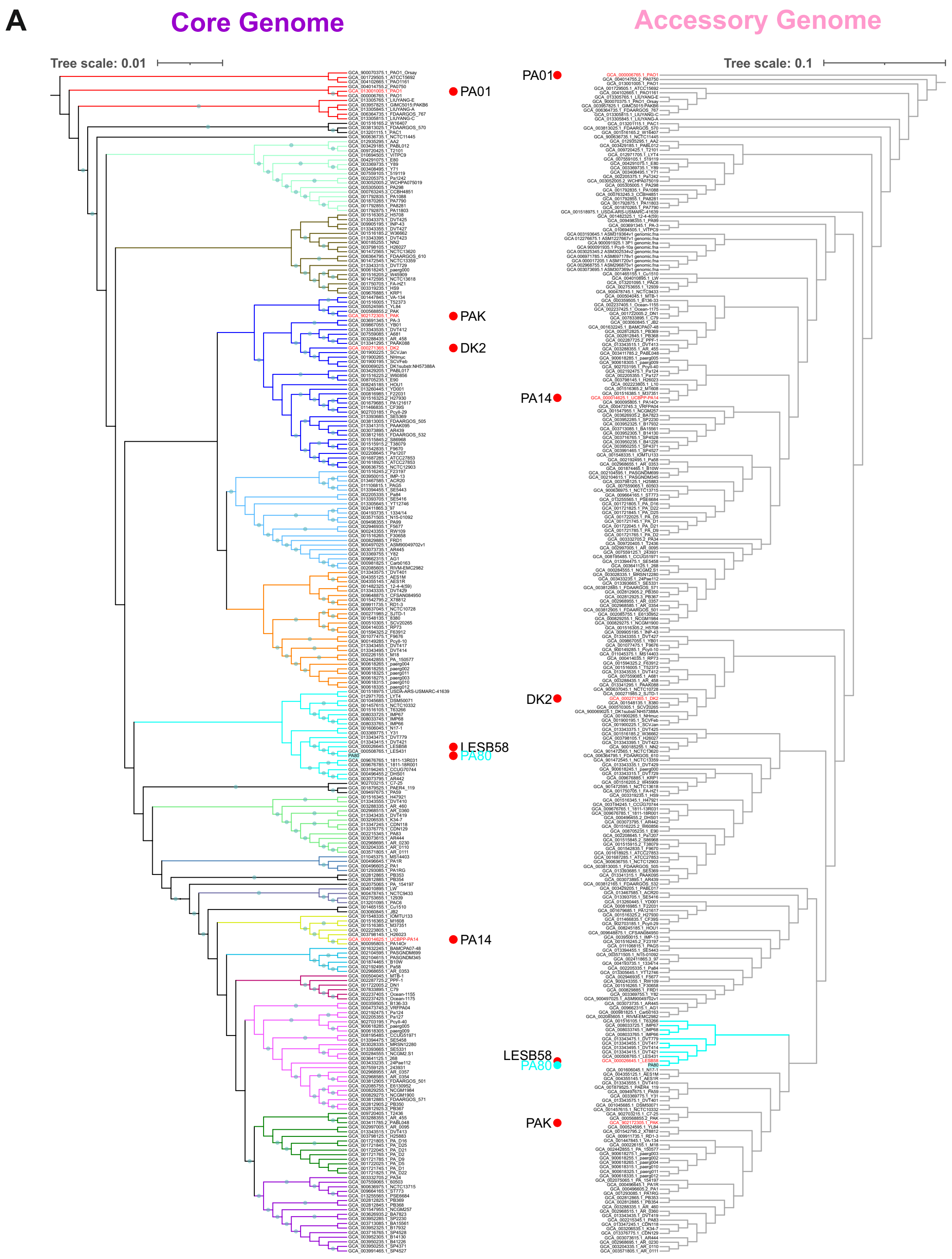

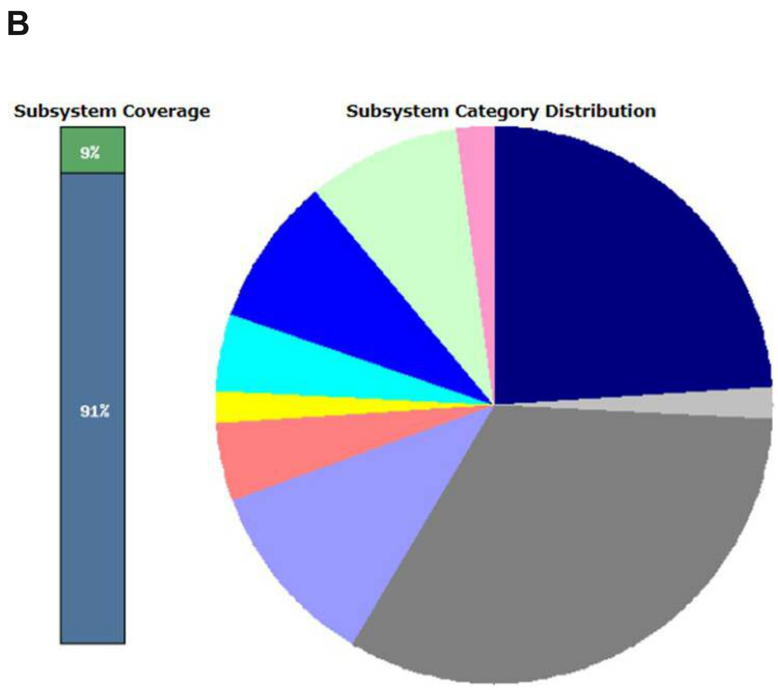

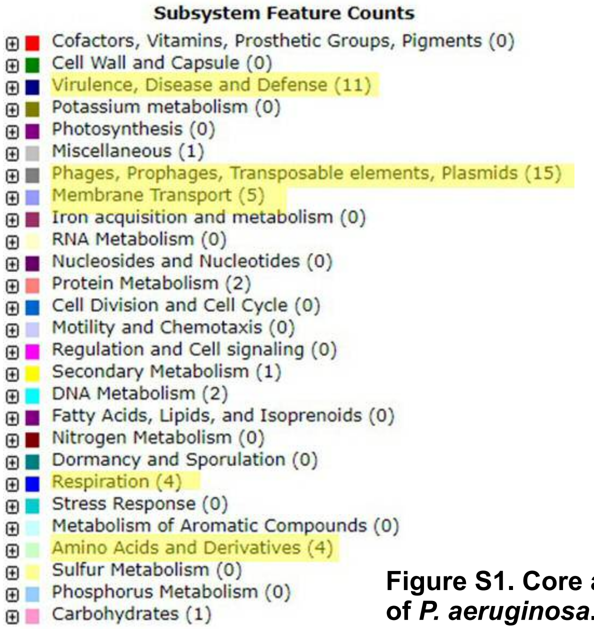

Figure S1. Core and Accessory Genome Phylogeny of *P. aeruginosa*. (**A**) Maximum likelihood cladogram of both core and accessory genomes. (**B**) Subsystem feature counts of *P. aeruginosa* PA80.

Table S1 List of gene variations in PA80 relative to PAO1 in common pathoadaptive genes.

Gene	Function	SNPs	Type of mutation
phoB	PhoB controls expression of various genes involved with cytotoxicity through modulation of QS systems in P-depleted conditions	0	no mutation
algR	represses the Rhl QS in a biofilm specific manner	0	no mutation
anr	induces expression of genes through synergy with *las* and *rhl* QS in anoxic conditions and has also shown to regulate virulence genes in LasR mutants.	4	synonymous
dksA	inhibits QS virulence factor productions by repressing transcription of *rhlI*	0	no mutation
gacA	*gacA* positively controls QS through activation of the Rhl system	3	synonymous
gacS	the GacS/GacA system positively controls QS by controlling the expression of *rsmY* and *rsmZ*	7	synonymous
mvaT	controls arginine metabolism, pyocyanin synthesis and prophage activation in PAO1	1	synonymous
qscR	QscR represses some QS controlled genes with *qscR* mutants being hypervirulent	1	synonymous
qteE	represses the expression of several *las* and *rhl*-dependent target genes by independently reducing LasR and RhlR protein stability	3	synonymous
relA	influences the PQS system with *relA* mutants showing decreased elastase production and reduced *P. aeruginosa* virulence in *in vivo* model	9	synonymous
rpoN	RpoN positively regulates the expression of *rhlI* and *pqsR* in PAO1	2	synonymous
rpoS	it can govern expression of genes required for the synthesis of pyocyanin, exotoxin, LasA and LasB elastases etc.	3	synonymous
rpoD	it recognizes many promoters and controls expression of housekeeping genes	3	synonymous
rsaL	RsaL represses *lasI* transcription and functions in opposite to LasR by counterbalancing C12-HSL concentrations	0	no mutation
rsmA	RsmA positively controls swarming and extracellular production of rhamnolipid and lipase	1	synonymous
vfr	it is global regulator and induces expression of the *lasR* promoter and virulence gene expressions.	1	synonymous
mvfR	involved in production of QS signal molecules and can regulate multiple QS controlled genes without affecting the *las* or the *rhl* QS systems.	1	synonymous
ampR	AmpR plays a dual role, positively regulating the *ampC*, *lasB*, and *rhlR* expression levels and negatively regulating the *poxB*, *lasA*, *lasI*, and *lasR* expression levels	1	synonymous
suhB	positive regulator of multiple genes important for *P. aeruginosa* virulence and pathogenesis	6	synonymous
algU	responsible for transcription of the alginate biosynthesis operon leading to mucoidity and robust biofilms in CF patients	0	no mutation
mexR	repressor of the mexAB-oprM multidrug efflux operon	1	R83C
pilR	transcriptional regulator of piliation- associated with virulent phenotype motility	9	E318D
lasR	master regulator of quorum sensing circuit, involved with expression of factors which gives bacteria many of its pathogenic trait	0	no mutation
lasI	produces a key autoinducer signal molecule C12HSL which positively regulates QS	0	no mutation
rhlR	regulator of the *rhl* mediated QS	-	null mutation
rhlI	produces a key autoinducer signal molecule C4-HSL which positively regulates QS	8	S62G, D83E
phzS	pyocyanin biosynthesis protein	19	Q154L, R180G, D256N
fur	coordinates the expression of several genes in iron depleted conditions	1	synonymous

*mexT**	Involved with increased antimicrobial resistance and repression of QS	14	synonymous
*mexS**	Involved with increased antimicrobial resistance through activation of the mexEF-oprN operon	4	D249N, M271I
himA	important for efficient transcription of the *algD*	3	H41Q
ptxR	transcriptional activation of *toxA* but has also shown to reduce PQS expression and pyocyanin production	3	S311G
algW	involved in cleavage of MucA which is a AlgU repressor	7	D386N
mucA	Inactivation of *mucA* results in constitutive expression of alginate biosynthesis gene	1	synonymous
fleQ	major (positive) regulator of flagellar genes	13	synonymous
pmrA	modulates resistance to cationic antimicrobial peptides	5	L71R, D104Y
argR	essential for induction of operons involved with arginine utilization.	2	synonymous
pvdS	involved in expression of pyoverdin and exotoxin A; also functions as iron starvation sigma factor	0	no mutation
cbrB	functions in carbon catabolism with mutants unable to utilize several C and N sources and suffer from impaired biofilm and stress tolerance	1	V142A
phoP	the phoP/phoQ two component regulatory system controls cytotoxicity and inflammation	1	synonymous
phoR	part of the phoB-phoR two component system, involved with induction of virulence genes in low phosphate conditions	4	Q58H
cysB	negatively affects the transcription of *pqsR* and PQS signal production	5	synonymous
exsA	transcriptional activator of the Type III secretion system	2	T262A
pprB	positively regulates transcription of type I secretion system, components, fimbriae, and type IV pili	9	S129N, R179K, P191S
psrA	controls the synthesis quinolone signal via repression of the FadE homolog	0	no mutation
roxR	activate expression of the cyanide-insensitive terminal oxidase	0	no mutation
np20	transcriptional regulator of the zinc uptake system in *P. aeruginosa*	4	synonymous
narL	regulatory gene involved with nitrate respiration in anaerobic conditions	1	synonymous
PA4851	hypothetical protein	15	A21V, V63A, I285V
PA1520	probable transcriptional regulator	1	synonymous
nfxB	*nfxB* mutant was impaired in all forms of motility as well as in the production of siderophores, rhamnolipid, secreted protease, and pyocyanin	4	Synonymous
vqsR	activated by *las* QS and plays essential role in acyl-HSL production and the expression of many quorum-controlled genes	1	synonymous
